# Supplementary material for: Programmed Cell Death Alterations Mediated by Synthetic Indole Chalcone Resulted in Cell Cycle Arrest, DNA Damage, Apoptosis and Signaling Pathway Modulations in Breast Cancer Model
Source: Pharmaceutics. 2022 Feb 24;14(3):503. doi: 10.3390/pharmaceutics14030503 (PMC8953149; doi:10.3390/pharmaceutics14030503)
Supplement: Supplementary file 1 [file pharmaceutics-14-00503-s001.zip › pharmaceutics-1551313-supplementary.pdf]

pharmaceutics

Supplementary Materials: Programmed Cell Death Alterations Mediated by Synthetic Indole Chalcone Resulted in Cell Cycle Arrest, DNA Damage, Apoptosis and Signaling Pathway Modulations in Breast Cancer Model

Radka Michalkova, Martin Kello, Zuzana Kudlickova, Maria Gazdova, Ladislav Mirossay, Gabriela Mojzisova and Jan Mojzis

1. The structure of compound was confirmed by ^{1}H, ^{13}C NMR, IR and HRMS spectroscopy:

^{1}H NMR (DMSO-d6, 600 MHz): 12.11 (s, 1H, NH), 7.88 (dd, 1H, J 15.4, 1.5 Hz, H-3), 7.71 (td, 1H, J 7.6, 1.8 Hz, H-6''), 7.66 (d, 1H, J 7.8 Hz, H-4'), 7.59 (tdd, 1H, J 8.1, 5.2, 1.8 Hz, H-4''), 7.35 – 7.32 (m, 3H, H-7', H-5'', H-3''), 7.16 (td, 1H, J 7.5, 1.1 Hz, H-6'), 7.11 (td, 1H, J 7.7, 1.1 Hz, H-5'), 7.06 (dd, 1H, J 15.4, 2.2 Hz, H-2), 4.36 (t, 2H, J 6.5 Hz, OCH2CH2CH3), 1.82 (qt, 2H, J 7.4, 6.5 Hz, OCH2CH2CH3), 1.01 (t, 3H, J 7.4 Hz, OCH2CH2CH3).

^{13}C NMR (DMSO-d6, 150 MHz): 187.3 (C, d, JCF 2.2 Hz, C-1), 159.9 (C, d, JCF 249.1 Hz, C-2''), 156.9 (C, C-2'), 136.9 (CH, C-3), 133.0 (CH, JCF 8.7 Hz, C-4''), 132.0 (C, C-7'a), 130.3 (CH, d, JCF 3.2 Hz, C-6''), 128.2 (C, d, JCF 14.4 Hz, C-1''), 125,0 (C, C-3'a), 124.7 (CH, d, JCF 3.2 Hz, C-5''), 121.8 (CH, C-5'), 121.2 (CH, C-6'), 118.4 (CH, C-4'), 116.4 (CH, d, JCF 23.0 Hz, C-3''), 115.7 (CH, d, JCF 5.5 Hz, C-2), 111.5 (CH, C-7'), 93.8 (C, C-3'), 73.0 (CH2, OCH2CH2CH3), 22.1 (CH2, OCH2CH2CH3), 10.0 (CH3, OCH2CH2CH3).

IR (KBr) νmax 1624, 1607, 1579, 1561, 1517, 1484, 1460, 1339, 1280, 1229, 1211, 1203, 1065 cm−1.

HRMS: m/z [M+H]+: 324.13989 for C20H19NO2F (calcd. 324.13943).

2. Densitometric analyses of Western blot

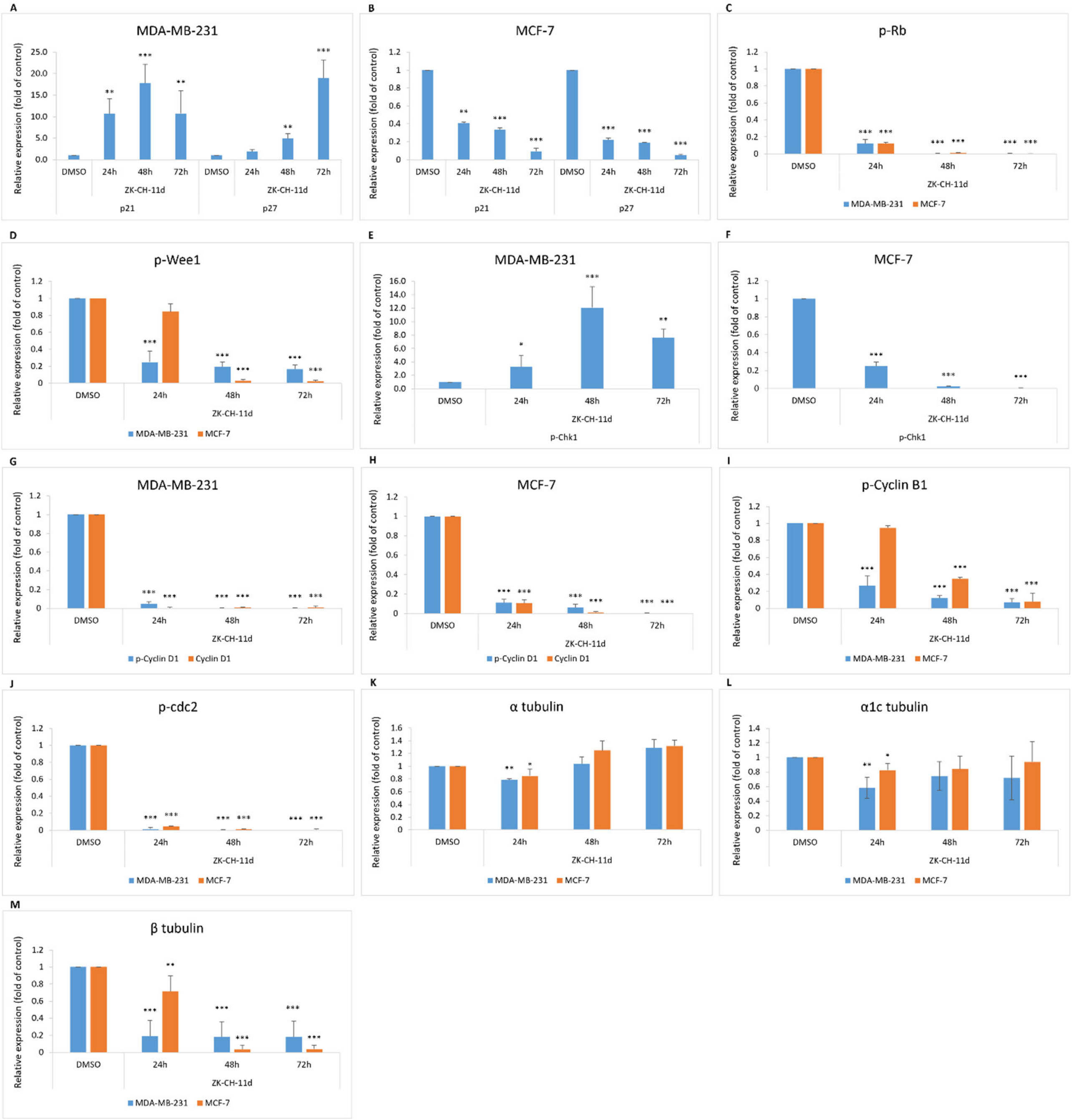

Figure S1. Densitometric analysis of western blot assay: relative expression of p21, p27 (**A**, **B**), p-Rb (**C**), p-Wee1 (**D**), p-Chk1 (**E**, **F**), p-Cyclin D1, Cyclin D1 (**G**, **H**), p-Cyclin B1 (**I**), p-cdc2 (**J**), *α* tubulin (**K**), *α* 1c tubulin (**L**) and β tubulin (**M**) in MDA-MB-231 and MCF-7 cells after ZK-CH-11d treatment. Statistical significance: * $p < 0.05$, ** $p < 0.01$, *** $p < 0.001$ vs. vehicle.

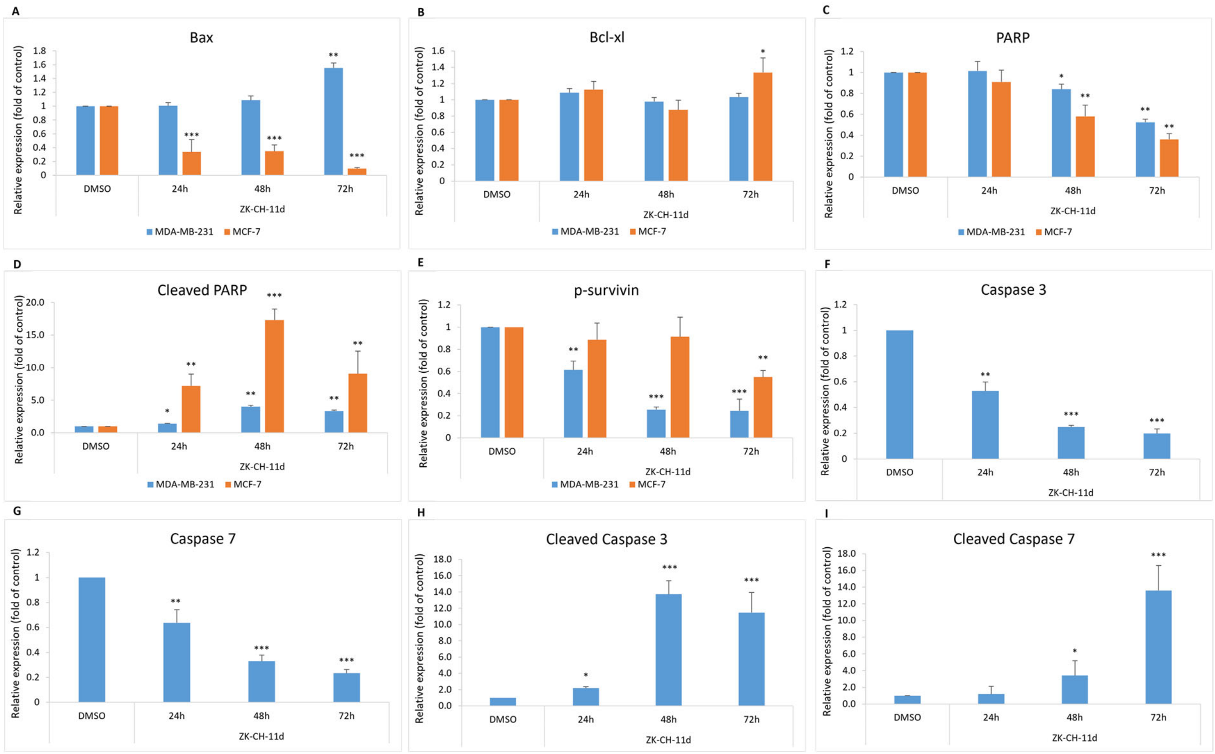

Figure S2. Densitometric analysis of western blot assay: relative expression of Bax (**A**), Bcl-xl (**B**), PARP (**C**), cleaved PARP (**D**), p-survivin (**E**), Caspase 3 (**F**) and Caspase 7 (**G**) and their cleaved forms (**H**, **I**) in MDA-MB-231 and MCF-7 cells after ZK-CH-11d treatment. Statistical significance: * $p < 0.05$, ** $p < 0.01$, *** $p < 0.001$ vs. vehicle.

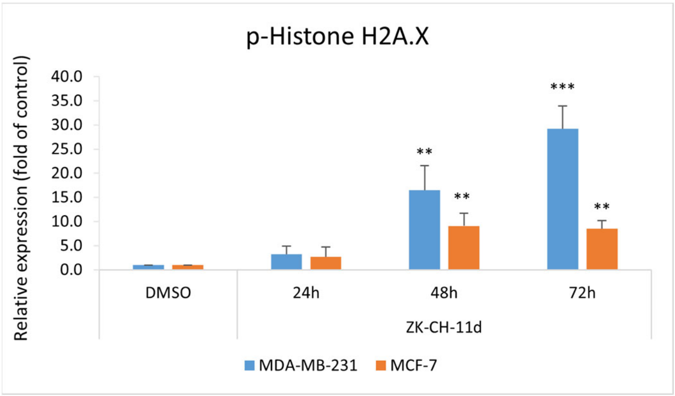

Figure S3. Densitometric analysis of western blot assay: relative expression of p-histone H2A.X in MDA-MB-231 and MCF-7 cells after ZK-CH-11d treatment. Statistical significance: ** $p < 0.01$, *** $p < 0.001$ vs. vehicle.

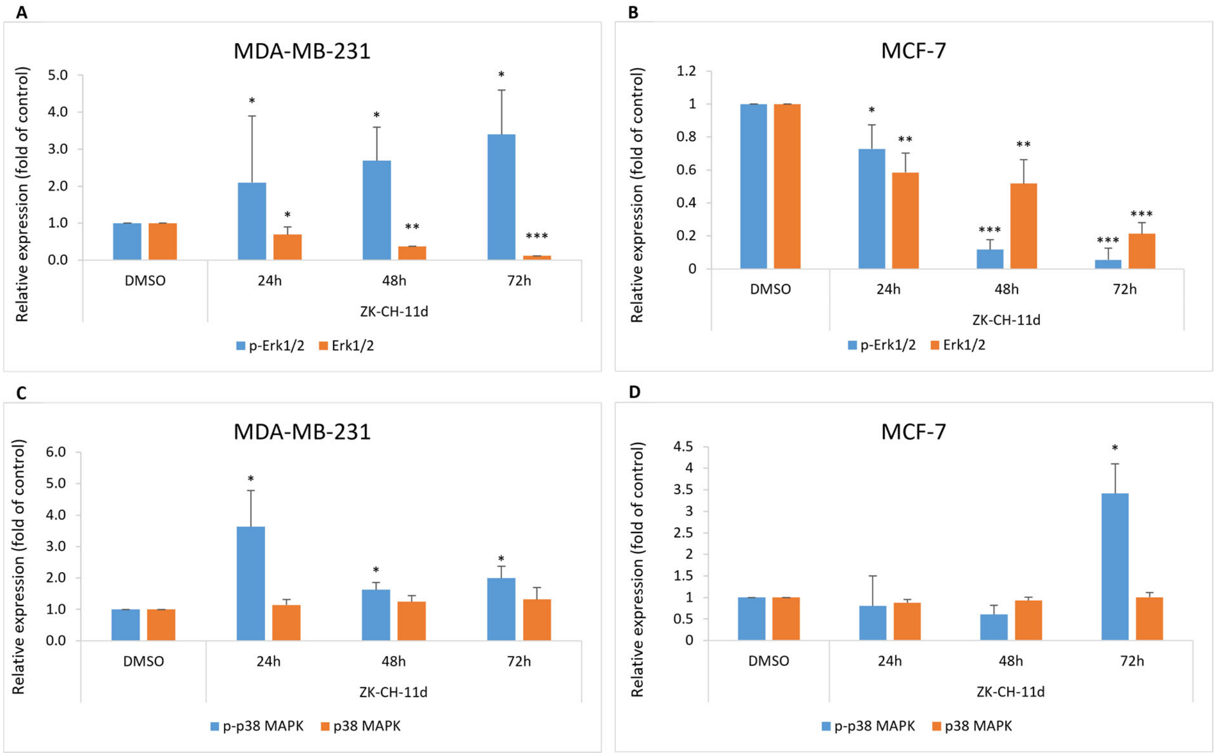

Figure S4. Densitometric analysis of western blot assay: relative expression of p-Erk1/2 and Erk1/2 (**A**, **B**) and p-p38 MAPK and p-38 MAPK (**C**, **D**) in MDA-MB-231 and MCF-7 cells after ZK-CH-11d treatment. Statistical significance: * $p < 0.05$, ** $p < 0.01$, *** $p < 0.001$ vs. vehicle.

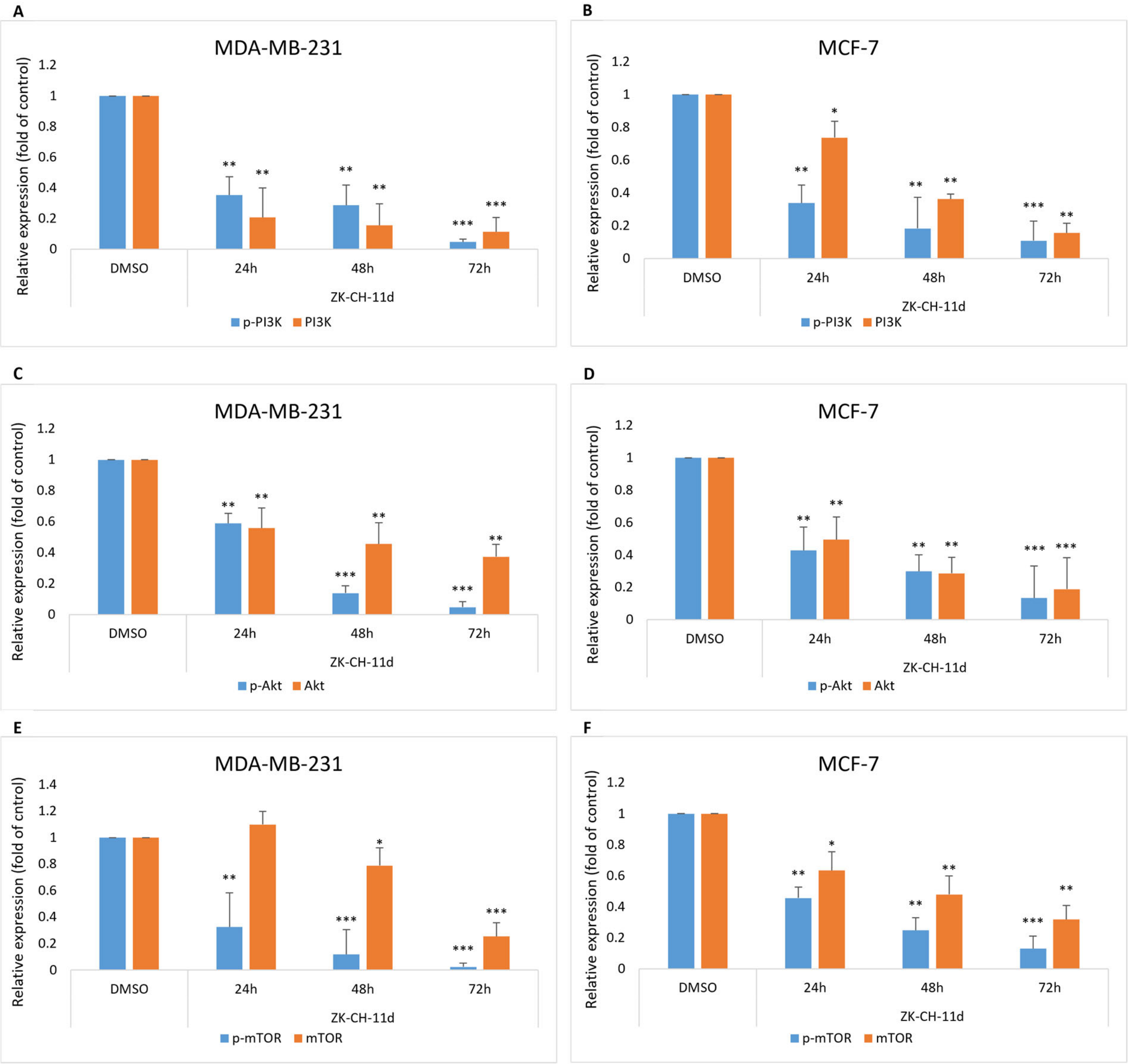

Figure S5. Densitometric analysis of western blot assay: relative expression of p-PI3K and PI3K (**A**, **B**), p-Akt and Akt (**C**, D) and p-mTOR and mTOR (**E**, **F**) in MDA-MB-231 and MCF-7 cells after ZK-CH-11d treatment. Statistical significance: * $p < 0.05$, ** $p < 0.01$, *** $p < 0.001$ vs. vehicle.

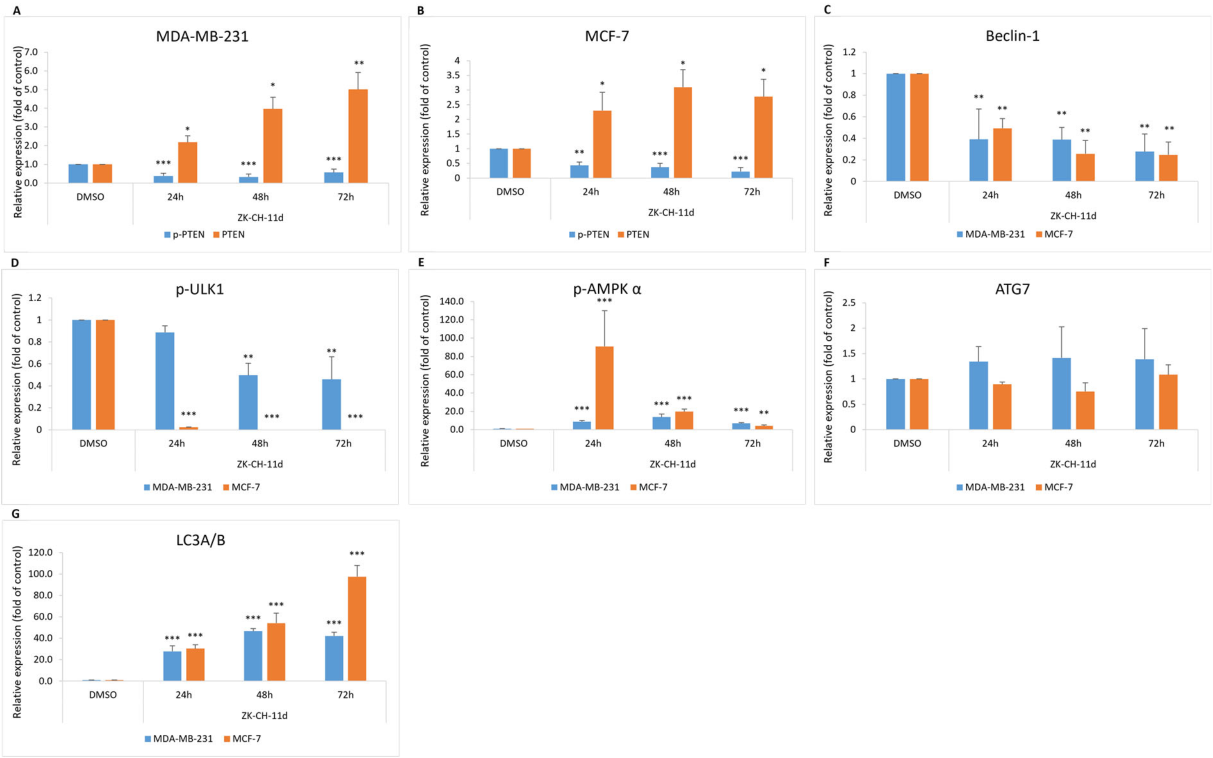

Figure S6. Densitometric analysis of western blot assay: relative expression of p-PTEN (**A**, **B**), PTEN (**A**, **B**), beclin-1 (**C**), p-ULK1 (**D**), p-AMPK*α* (**E**), ATG7 (**F**) amd LC3A/B (**G**) in MDA-MB-231 and MCF-7 cells after ZK-CH-11d treatment. Statistical significance: * $p < 0.05$, ** $p < 0.01$, *** $p < 0.001$ vs. vehicle.
